# The Impact of Social Media Use on Sleep and Mental Health in Youth: a Scoping Review

**DOI:** 10.1007/s11920-024-01481-9

**Published:** 2024-02-08

**Authors:** Danny J. Yu, Yun Kwok Wing, Tim M. H. Li, Ngan Yin Chan

**Affiliations:** Li Chiu Kong Family Sleep Assessment Unit, Department of Psychiatry, Faculty of Medicine, The Chinese University of Hong Kong, Shatin, Hong Kong SAR, China

**Keywords:** Social media use, Sleep, Mental health, Youth, Adolescent

## Abstract

**Purpose of Review:**

Social media use (SMU) and other internet-based technologies are ubiquitous in today’s interconnected society, with young people being among the commonest users. Previous literature tends to support that SMU is associated with poor sleep and mental health issues in youth, despite some conflicting findings. In this scoping review, we summarized relevant studies published within the past 3 years, highlighted the impacts of SMU on sleep and mental health in youth, while also examined the possible underlying mechanisms involved. Future direction and intervention on rational use of SMU was discussed.

**Recent Findings:**

Both cross-sectional and longitudinal cohort studies demonstrated the negative impacts of SMU on sleep and mental health, with preliminary evidence indicating potential benefits especially during the COVID period at which social restriction was common. However, the limited longitudinal research has hindered the establishment of directionality and causality in the association among SMU, sleep, and mental health.

**Summary:**

Recent studies have made advances with a more comprehensive understanding of the impact of SMU on sleep and mental health in youth, which is of public health importance and will contribute to improving sleep and mental health outcomes while promoting rational and beneficial SMU. Future research should include the implementation of cohort studies with representative samples to investigate the directionality and causality of the complex relationships among SMU, sleep, and mental health; the use of validated questionnaires and objective measurements; and the design of randomized controlled interventional trials to reduce overall and problematic SMU that will ultimately enhance sleep and mental health outcomes in youth.

## Introduction

Youth population, which typically refers to individuals between the ages of 15 and 24, experience substantial changes in their neurobiology, physical development, behavior, and emotions, making it a vulnerable stage for the development of both sleep and mental health problems [1–3]. In Hong Kong, approximately 64.5% of adolescents sleep less than 8 h during weekdays [4] and 29.2% have reported insomnia symptoms [5]. Both cross-sectional and longitudinal studies have demonstrated that sleep loss and disturbances in youth lead to significant personal distress, increase risk of psychiatric illnesses, and risky behaviors such as drug abuse and dangerous driving [5, 6]. In addition to sleep disturbances, mental health problems are highly prevalent among the youth population. Evidence suggested that nearly 75% of psychiatric illnesses have their age onset during adolescence [7, 8].

There are multiple risk factors that commonly contribute to sleep and mental health problems, including being female, heavy school workload, physical inactivity, and worse general health [9]. Recently, a growing number of studies indicate that social media use (SMU) is associated with both sleep and mental health problems in youth [10•]. In particular, identity development and peer acceptance during adolescence are important developmental needs, at which social media may apparently serve as a convenient means to meet these needs. A previous study reported that over 80% of adolescents (16–19 years) use electronic devices near bedtime [11]. On the other hand, excessive SMU can have detrimental health effects [12, 13], and contribute to various negative repercussions such as cyberbullying [14], gender stereotypes [15], self-objectification [16], and exposure to inappropriate content, such as unsolicited violent and sexual contents [17]. The effect becomes more prominent in young people who are considered as digital native [18]. Nevertheless, SMU also comes with some potential benefits [19] such as increased self-esteem [20], increased social capital [21], identity presentation and sexual exploration [22], and social support [23].

Despite the emerging evidence supporting the link among SMU, sleep, and mental health, the relationship and directionality are complex and inconsistent. For example, two recent studies did not find significant associations among SMU, sleep, and mental health [24, 25•]. Nonetheless, a US study reported that greater SMU was significantly associated with sleep disturbances [26], and some also reported bidirectional relationship at which poor sleepers tend to use electronic devices as a sleep aid [27]. In general, it is believed that youth are at a higher risk of experiencing the negative impacts of SMU as they are more susceptible to peer pressure and fear of missing out (FOMO). FOMO refers to the perception of missing out on enjoyable experiences, followed up with a compulsive behavior to maintain these social connections with others to avoid being excluded from those experiences [28–30]. Hence, understanding the association and directionality among SMU, sleep, and mental health is crucial for developing public health strategies on how to cultivate healthy SMU habits and develop effective interventions targeting inappropriate and excessive SMU.

**Fig. 1:**
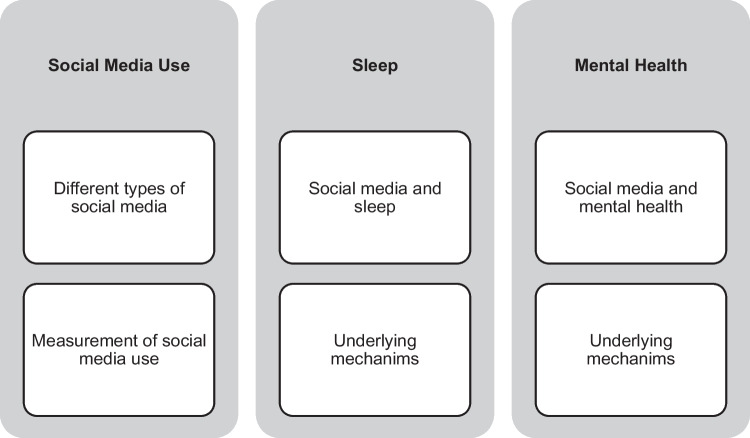
Structure of the scoping review

**Fig. 2:**
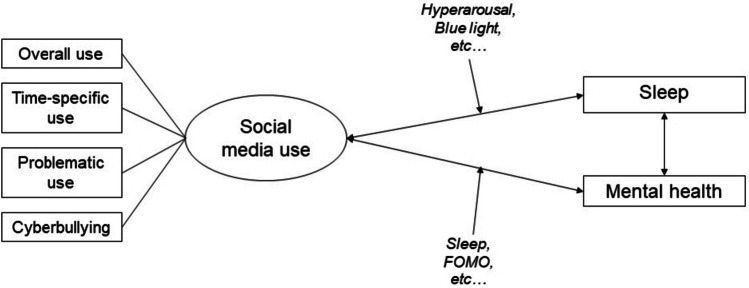
Potential pathways of social media use on sleep and mental health

This scoping review summarized recent studies on SMU, sleep, and mental health in youth (Fig. 1) and explored the potential underlying mechanisms of how SMU affects sleep and mental health in youth (Fig. 2). Finally, we have put forward several potential avenues for future research and recommendations in this area. Search terms including #adolescent, #social media, #sleep, and #mental health were used to search for relevant studies that were published between January 2020 and July 2023 in MEDLINE. A summary of the study attributes, such as the authors, the country/region where the study was conducted, study design, the number of participants, sample age range, characteristics, as well as the measures used to assess SMU, sleep, and mental health are listed in Table 1.

**Table 1:** Summary of studies on social media use (SMU), sleep, and mental health

**Author**	**Country/region**	**Study design**	**Sample size**	**Sample characteristics**		**SMU measures**	**Sleep and mental health measures**	**Main findings**
				Age	Female %			
Barthorpe et al.	UK	Cross-sectional	4032	13–15	55.2	SMU (time use diary)	Self-harm (single question) Depressive symptoms (short mood and feelings questionnaire, SMFQ) Self-esteem (5-item shortened Rosenberg scale)	A greater amount of time spent on social media was associated with increased risk of self-harm and depression and lower levels of self-esteem in females
Bailey et al.	Australia	Cross-sectional	371	16–25	70.4	SMU (self-report questionnaire)	Mental health (Depression Anxiety Stress Scales (DASS-21)) Diagnosis of mental health problem (single question) Impact of COVID-19 on mental health (single question)	High levels of daily SMU were associated with poorer mental health Young people use social media to seek support for suicidal thoughts and self-harm
Berfeld and Bulck	US	Cross-sectional	410	12–18	54.3	Affordances of social media (self-report questionnaire) Nighttime SMU (self-report questionnaire) Problematic SMU (the Bergen Facebook Addiction Scale) Adverse SMU (self-report questionnaire)	Sleep displacement (sleep diary) Pre-sleep arousal (the Pre-Sleep Arousal Scale) Sleep quality (the Pittsburgh Sleep Quality Index, PSQI)	Use of Snapchat but not Instagram predicted a later bedtime Social media affordances were associated with at least one sleep parameter Being the second most viewed content, viewing post of celebrities had no relationship with sleep
Brailovskaia et al.	Russia	Cross-sectional	1123	18–29	100	SMU (time tracked by specific applications, and self-report questionnaire) Problematic SMU (the brief version of the Bergen Social Media Addiction Scale, BSMAS)	Daily stress (the Brief Daily Stressors Screening Tool, BDSS-Tool) Depression and anxiety symptoms (the Depression Anxiety Stress Scales 21 (DASS-21)) Positive mental health (the unidimensional Positive Mental Health Scale, PMH-Scale) Lifetime suicide-related outcomes (the Suicidal Behaviors Questionnaire-Revised, SBQ-R)	SMU was associated with low mental health, and increased suicide-related outcomes
Buda et al.	Lithuania	Cross-sectional (representative sample)	4191	13.9	49.1	Problematic SMU (the nine-item Social Media Disorder Scale)	Sleep problems (difficulties in getting to sleep, and tiredness in the morning) (self-report questionnaire)	Increased SMU is associated with worse sleep quality
Burnell et al.	US	Cross-sectional	388	13.37	50	Daily digital technology use (total amount of messages sent and duration spent online and on phone)	Subjective sleep parameters (sleep duration, sleep quality) (sleep diary) Objective sleep parameters (Jawbone wearable device)	Greater non-academic digital technology use was associated with shorter subjective and objective sleep duration, and later bedtime
Chao et al.	China	Cross-sectional	1346	14.97	51.8	Short video platform use (self-report questionnaire) Short video addiction (the short version of smartphone addiction scale, SAS-SV)	Sleep quality and duration (self-report questionnaire) Social anxiety (the social interaction anxiety scale, SIAS-6) Psychological distress (the 21-item Depression Anxiety Stress Scale, DASS-21) Loneliness (the UCLA loneliness scale, ULS-4)	Significant association between short video use, sleep quality, and mental health conditions was observed
Charmaraman et al.	US	Cross-sectional	772	11–15	N/A	SMU (self-report questionnaire)	Sleep (sleep duration and bedtime) (self-report questionnaire)	Self-report SMU behaviours were associated with negative sleep outcomes
Fardouly et al.	Australia	Cross-sectional	528	10–12	49.1	SMU (self-report questionnaire)	Body satisfaction (the Body Esteem Scale for Adolescents and Adults) Eating pathology (the Children’s Eating Attitude Test) Depressive symptoms (the Short Mood and Feelings Questionnaire) Social anxiety (the Spence Children’s Anxiety Scale)	Users of YouTube, Instagram, and Snapchat reported more body image concerns and eating pathology than non‐users Social media appearance comparisons are associated with negative mental health among preadolescents
Friebel et al.	UK	Cross-sectional	101	19.7	N/A	Emotional investment in social media (the Social Media Use Integration Scale) SMU in the last hour before sleep	Sleep quality (the Pittsburgh Sleep Quality Index, PSQI) Objective sleep parameters (actigraphy) Depressive symptoms (the 10-item Centre for the Epidemiologic Studies of Depression Short Form, CESD-10)	Bedtime SMU may not be detrimental to sleep in youth Youth with increased levels of depressive symptoms are at higher risk of experiencing negative impacts of bedtime SMU
Gaya et al.	Spain	Cross-sectional	1101	12–17	54.2	SMU (self-report questionnaire, and ecological momentary assessment)	Sleep duration and sleep-related problems (self-report questionnaire, the BEARS scale	A relationship between cell phones, playing video games, and use of social networks and sleep problems
Gumport et al.	US	Cross-sectional	176	10–18	58	SMU (self-report questionnaire and ecological momentary assessment	Objective sleep parameters (total sleep time, bedtime, and sleep onset latency) (actigraphy) Emotional health (Ecological Momentary Assessment)	Technology use was associated with increased sleep onset latency, but better emotional health
Hamilton et al.	US	Cross-sectional	93	12–17	100	SMU (self-report questionnaire)	Perceived sleep (sleep quality and sleep duration) (self-report questionnaire) Depressive symptoms (6-item Patient Health Questionnaire) Anxiety symptoms (Generalized Anxiety Disorder-7, GAD-7)	During COVID-19 pandemic, higher levels of SMU and lower levels of physical activity are associated with later sleep timing
Hasan Beyari	Saudi Arabia	Cross-sectional	385	18–24	44.7	SMU (self-report questionnaire)	Mental health (self-report questionnaire)	Social media has a significant negative impact on the mental health of Saudi Arabian youth
Henzel and Håkansson	Sweden	Cross-sectional	182	16–24	N/A	Social media addiction (the Bergen Social Media Addiction Scale, BSMAS; the Problematic and Risky Internet Use Screening Scale-3, PRIUSS) SMU over the past 12 months (self-report questionnaire)	Mental health (the Kessler Psychological Distress Scale, Kessler-6) Addictive disorder (the Gaming Addiction Scale, GAS; The Problem Gambling Severity Index, PGSI)	Bidirectional relationship was observed between problematic SMU and psychological distress, as well as alcohol abuse
Khan et al.	Europe and North America	Cross-sectional (representative sample)	195,668	13.6	51	Self-report discretional time spent watching TV, electronic gaming, and computer use	Difficulty in falling sleep (self-report questionnaire)	Higher levels of recreational screen use were associated with sleep-onset difficulties
Kortesoja et al.	Finland	Cross-sectional	1084	15–20	45.7	Evening and late-night SMU (self-report questionnaire) SMU after lights out (self-report questionnaire)	Sleep quality (the Pittsburgh Sleep Quality Index, PSQI) Chronotype (the Morningness-Eveningness Questionnaire) Bedtime, wake-up time, and sleep duration (self-report questionnaire) Insufficient sleep (self-report questionnaire)	Youth with evening chronotype are at greater risks of late-night media use, which negatively affect sleep
Li et al.	Hong Kong	Cross-sectional	3455	14.9	55.7	Electronic media use (self-report questionnaire)	Sleep (the Insomnia Severity Index, ISI) Chronotype preference (the reduced Horne and Östberg Morningness and Eveningness Questionnaire, rMEQ) Sleep deprivation Social jetlag Emotional difficulties (the Strengths and Difficulties Questionnaire, SDQ)	Bedtime electronic media use of computers, electronic game consoles, and phones contributed to increased risks of sleep, circadian problems and mental health issues in adolescents
Liu et al.	China	Cross-sectional	4852	13.8	51.5	Problematic internet use (the 26-item Chinese Internet Addiction Scale-Revised)	Sleep quality (the Chinese version of the 8-item Athens Insomnia Scale)	Problematic internet use was associated with higher level of insomnia severity
Michelle O’Reilly	UK	Cross-sectional	54	11–18	44.4	N/A	Mental health (focus group discussion)	SMU confers negative impacts on mental health of adolescents, although there was acknowledgement of potential benefits
Nagata et al.	US	Cross-sectional (Representative sample)	11,878	12.0	48.8	Bedtime screen usage (self-report questionnaire) Overall screen usage (self-report questionnaire)	Sleep disturbance (self-report questionnaire) Presence of sleep disorders (26-item parent-report questionnaire)	Engaging in electronic devices use was associated with overall sleep disturbance, sleep onset and daytime sleepiness
Nissim et al.	Europe and North America	Cross-sectional (Representative sample)	86,542	13–15	50.9	Intense SMU (EU Kids Online Survey) Problematic SMU (the Social Media Disorder Scale)	Sleep duration (self-report questionnaire)	Across 18 countries, both intense and problematic SMU were associated with shorter sleep duration, later bedtimes and greater social jetlag
Tao and Fisher	US	Cross-sectional	407	15–18	82.3	Hours of SMU (self-report questionnaire) Social media intergroup contact (the Developmental Intergroup Contact Measure, DICM) Social media racial justice civic engagement (the Online Civic Engagement Behaviour Construct) Social media racial discrimination (the Online Victimization Scale)	Depressive symptoms (the 20-item Center for Epidemiologic Studies Depression Scale, CES-D) Anxiety symptoms (the 7-item Generalized Anxiety Disorder, GAD-7) Alcohol use disorder (Alcohol Use Disorders Identification Test (AUDIT)) Illicit drug use and problems (self-report questionnaire)	SMU and racial discrimination are associated with poor mental health and drug use problem
Valero et al.	UK	Cross-sectional	2232	18.0	51.1	Problematic digital technology use (the Compulsive Internet Use Scale)	Sleep quality (the Pittsburgh Sleep Quality Index, PSQI) Loneliness (the UCLA Loneliness Scale, version 3) Depression and anxiety symptoms (structured clinical interview)	Problematic use of technology was associated with poor sleep quality, after controlling for loneliness, depressive and anxiety symptoms, neighbourhood disorder, sex, and maternal insomnia
Woods and Scoot	UK	Cross-sectional	467	11–17	N/A	Overall and night-time-specific SMU (self-report questionnaire)	Anxiety and depression (the Hospital Anxiety and Depression Scale (HADS)) Self-esteem (the Rosenberg Self-Esteem Scale, RSES) Emotional investment in social media (the Social Media Use Integration Scale) Sleep quality (the Pittsburgh Sleep Quality Index (PSQI))	Overall and night-time-specific SMU were associated with lower self-esteem and increased anxiety and depression
Xie et al.	Macau	Cross-sectional	212	15–24	51.9	Screen-based electronic media device usage (self-report questionnaire)	Sleep quality (the Pittsburgh Sleep Quality Index (PSQI))	A J-shape relationship was observed between TV, computer, mobile phone use and sleep quality
Beeres et al.	Sweden	Cohort	3501	14–15	51.5	SMU (self-report questionnaire)	Mental health (Strength and Difficulties Questionnaire (SDQ))	Higher use of social media was associated with more symptoms of mental health problems. No longitudinal association was found
Eijnden et al.	Netherlands	Cohort	2021	13.9	45.4	Frequency of SMU (self-report questionnaire) Problematic SMU (the Social Media Disorder (SMD))	Sleep quality (the 5-item Groningen Sleep Quality Scale (GSKS)) Bedtime (self-report questionnaire)	The intensity of SMU and problematic SMU delay bedtime of adolescents
Lee et al.	US	Cohort	5114	12–14	48.7	SMU (self-report questionnaire)	Mental health (the Global Appraisal of Individual Needs-Short Screener (GAIN-)SS)	Frequent SMU negatively affects mental health in this recent representative sample of US adolescents
Maksniemi et al.	Finland	Cohort	426	13–19	65.7	Active SMU (the Socio-Digital Participation Inventory)	Bedtime on schooldays (self-report questionnaire) Emotional exhaustion (the School Burnout Inventory)	No clear pattern between active SMU and bedtime across adolescence Active SMU was associated with emotional exhaustion
Plackett et al.	UK	Cohort (representative sample)	3228	10–15	51.4	SMU (self-report questionnaire)	Mental health (the Strengths and Difficulties Questionnaire (SDQ))	SMU was not longitudinally associated with poorer mental health 2 years later
Richardson et al.	Australia	Cohort	528	10–12	49	Time spent using technology (self-report questionnaire) Parental control of technology (self-report questionnaire)	Chronotype preference (the 10-item Children’s Morningness-Eveningness Scale (MESC)) School night sleep duration (self-report questionnaire) Daytime sleepiness (the 8 item Paediatric Daytime Sleepiness Scale (PDSS))	This current study suggested a bidirectional relationship between technology use and sleep in adolescents Parental restriction of technology use may not predict prospective changes in adolescent technology use, nor sleep, over time
Zhou, et al.	China	Cohort	2329	18.4	63.1	SMU (self-report questionnaire)	Mental health (self-report questionnaire)	Problematic internet use was reciprocally associated with mental health issues

## Overview of SMU

SMU refers to the act of engaging with online platforms specifically designed for social interaction, whereas electronic media use (or digital use, digital media, internet use, screen time) is a broader term that encompasses various forms of media delivered electronically, including but not limited to social media. In this scoping review, we focus on SMU.

Over the past decade, subjective measures have been the primary tool to investigate individual perceptions, opinions, or personal experiences of SMU. For example, a self-report scale was developed to assess compulsive use of social media and its severity [31]. Besides, there are several platform-specific scales for social media addiction features such as salience, mood modification, tolerance, withdrawal, conflict, and relapse. The Bergen Facebook Addiction Scale, for instance, focuses specifically on addiction to Facebook [32•], while the Bergen Social Media Addiction Scale has emerged to examine a broader scope, including social media platforms beyond Facebook [33•, 34]. Indeed, more researchers used general metrics to measure SMU across multiple platforms collectively such as the Social Media Disorder Scale which measures aspects of social media addiction features, such as preoccupation with social media, excessive time spent, withdrawal symptoms, and negative consequences [35–37]. Other SMU experiences are also captured including social comparison on social media and negative experiences such as bullying, FOMO, and extensive negative feedback [32•].

In addition, the duration and timing of SMU also have significant implications for sleep and mental health, as excessive or inappropriate use of social media at certain timing, for example at bedtime, can potentially contribute to negative biopsychosocial effects. The Socio-Digital Participation Inventory includes four items to measure the frequency of SMU on a seven-point frequency scale (1 = never, 2 = a couple of times a year, 3 = monthly, 4 = weekly, 5 = daily, 6 = multiple times a day, 7 = all the time) [25•]. The total time spent on SMU (in daytime and night-time) are usually captured by questionnaires and social media time use diary [38•]. In view of the limitation of self-reported measures, there has been a shift towards incorporating more objective measures in addition to subjective self-report scales. Increasing number of studies used time tracker via specific apps (installed on participants’ devices used for online activity) to reduce recall bias [33•]. Other objective features, such as the number of followers, likes, comments, shares, bookmarks, and total interactions, which can be retrieved from various social media platforms [39•] were also used to reflect social medica engagement. In addition, the content (e.g., educational vs non-educational) posted, read, and shared on social media platforms plays a significant role in shaping user experiences, engagement levels, and the overall impact of SMU. It is worth to note that no included studies attempted to measure multi-device SMU as it can be challenging due to the wide range of devices that people use to access social media platforms. Traditional research methods often rely on self-reporting, surveys, or tracking software installed on specific devices, which may not capture the full extent of multi-device usage. Some individuals may switch between multiple devices throughout the day, making it challenging to track their overall social media engagement accurately.

## Overview of Recent Studies

### Study Characteristics

A total of 33 studies were included in this scoping review, with 26 of them were cross-sectional in nature, indicating a snapshot overview of the relationship between SMU, sleep, and mental health. Moreover, only a few studies utilized representative samples [35, 36, 40, 41], as outlined in Table 1. It is also worth noting that the sample sizes varied significantly across studies, ranging from 54 to 195,668 participants.

### Measurement of SMU

In terms of the measurement of SMU, all studies used either self-developed questionnaires (e.g., “in a typical school week, how often do you check social media?” and “on a normal weekday, how many hours you spend on social medias, write blogs/read each people blogs, or chat online?”) or validated self-report questionnaires (e.g., the 26-item Chinese Internet Addiction Scale-Revised and the Online Civic Engagement Behavior Construct). Different dimensions of SMU were measured such as overall and night-time SMU, problematic SMU, emotional investment in social media, racial discrimination, and racial justice civic engagement on social media. Only a limited number of studies incorporated more reliable measurements such as ecological momentary assessment [42] and total message count [43].

### Measurement of Sleep and Mental Health

In terms of sleep outcome assessment, most of these studies employed subjective instruments such as sleep diary [32•], self-developed self-report questionnaires (e.g., “How many hours did you sleep over the past week?”) [43], and validated self-report questionnaires (e.g., the Pittsburgh Sleep Quality Index and the Insomnia Severity Index) [44, 45•] to measure different sleep outcomes including sleep quality, sleep duration, sleep displacement, bedtime, and sleep-onset latency. In addition to these subjective instruments, 3 studies have utilized objective devices such as actigraphy and other wearable devices to capture objective sleep data [43, 46, 47]. While for mental health aspects, depression and anxiety are the main outcomes. Most of the recent studies utilized standardized questionnaires such as the Short Mood and Feelings Questionnaire, the Depression Anxiety Stress Scales 21, and the Suicidal Behaviors Questionnaire-Revised to measure the symptoms of depression and anxiety.

## Synthesis of Recent Findings

### SMU and Sleep

Both longitudinal and cross-sectional studies tend to support the association between SMU and sleep disturbances (Table 1). A total of 17 cross-sectional studies observed an association between SMU and various sleep parameters in youth. Of these studies, a total of 16 reported a significant association between different dimensions of SMU (internet addition, duration of screen use, inappropriate time use (near bedtime), with one additionally measure parent control of technology) and poor sleep outcomes (both subjectively and objectively measured sleep parameters, such as bedtime, sleep-onset latency, sleep duration, and sleep quality) [31, 32•, 35, 36, 40, 42–44, 45•, 47–53]. Nevertheless, a study of 101 undergraduate students did not find that bedtime SMU was detrimental to sleep [46]. However, in the subgroup analysis, the authors found that youth with increased levels of depressive symptoms are at higher risk of experiencing negative impacts of bedtime SMU on sleep [46].

Among the three cohort studies, two indicated that higher levels of SMU predicted later bedtime and shorter sleep duration in youth after 1–2 years of follow-up [37, 54]. These studies revealed that both frequent and problematic use of SMU could result in later bedtime [37, 54]. In addition, Richardson and colleagues further found that SMU predicted greater daytime sleepiness in adolescence [54]. In addition, adolescents with evening chronotype preference and shorter sleep duration were found to have longer usage of social media, suggesting a potential bidirectional relationship between SMU and sleep duration [54]. Another cohort study conducted by Maksniemi and colleagues did not find a significant association between SMU and bedtime among 426 youth aged between 13 and 19 [25•]. Interestingly, subgroup analyses indicated that significant associations were only observed in early adolescence (at age 13 and 14), but not in middle (at age 14 and 15) nor late adolescence (at age 17 and 18) [25•]. This finding highlights the importance of considering the developmental stages of youth in order to unravel the complex relationship between SMU and sleep [55].

### SMU and Mental Health

A total of 9 cross-sectional studies examined the relationship between SMU and mental health [33•, 34, 38•, 39•, 56•, 57–60]. A greater amount of time spent on social media was associated with an increased risk of depression, self-harm, and lower self-esteem. On the other hand, adolescents who exhibited mental health issues tended to spend more time on social media platforms, suggesting a potential bidirectional relationship between SMU and mental health. However, it is important to point out that despite appealing hypotheses, actual effect size estimates of SMU on various mental health outcomes (e.g., self-esteem, life satisfaction, depression, and loneliness) were of small-to-medium magnitude as reported in previous meta-analytic studies, ranging from − 0.11 to − 0.32 [61•, 62].

Four longitudinal cohort studies reported mixed findings between SMU and mental health [41, 63, 64•, 65]. Two cohort studies conducted in the USA and China reported that frequent and problematic SMU were significantly associated subsequent mental health issues [64•, 65]. Interestingly, the authors identified substantial sex differences in the mental health trajectories, with only girls showing a deteriorating linear trend (*β* = 0.23, *p* < 0.05) [64•]. On the contrary, the other two longitudinal studies conducted in Sweden and UK reported that although frequent SMU was associated with increased levels of mental problems at a single timepoint, there was no longitudinal association [41, 63], which suggests that SMU may be only an indicator for mental health instead of a risk factor.

### SMU, Sleep, and Mental Health

A total of 7 studies measured both sleep and mental health outcomes [31, 45•, 46–48, 50, 59]. Five of these studies reported significant associations among SMU, sleep, and mental health outcome [31, 45•, 46, 50, 59]. It was reported that SMU was significantly associated with poor sleep quality and increased mental health issues [31, 45•, 46, 50, 59], and sleep was found to mediate the negative impacts of SMU on mental health and emotional symptoms in adolescents [45•]. Poor sleep was also shown to be significantly associated with mental health outcomes [31, 50, 59]. Furthermore, adolescents with higher level of depressive symptoms were at higher risk of experiencing negative impacts of bedtime SMU on sleep outcomes [46]. Indeed, these findings preliminarily unveiled the complex interplay among SMU, sleep, and mental health.

Nevertheless, it is essential to highlight that recent research has also recognized the positive impacts of SMU on mental health, particularly in the context of the COVID-19 pandemic, at which physical social interactions were significantly disrupted [56•, 58]. Adolescents in Australia and UK were found to use social media as an active coping strategy to relieve external stressors (e.g., exam pressure), to seek support for suicidal ideation or self-harm behavior, and to support others via social media [56•, 58].

## Discussion

This scoping review synthesized recent publications from the past 3 years that investigated the impact of SMU on sleep and/or mental health outcomes in youth. The majority of the studies provide supporting evidence for an association between SMU, poor sleep quality, and adverse mental health outcomes. Problematic SMU or addiction, as well as the duration of SMU, were identified as the most prevalent aspects of social media examined in the included studies. Sleep duration, bedtime, and insomnia emerged as the most commonly assessed sleep problems, while depression and anxiety were the most frequently measured mental health outcomes. However, it is important to note that despite the significant associations identified among these variables, the directionality of the relationship remains unclear in view of inconsistent findings across studies.

### Underlying Mechanism Between SMU and Sleep

Numerous mechanisms have been proposed to elucidate the relationship between social/digital media usage and sleep quantity and quality [66]. Hyperarousal, a core mechanism in explaining insomnia [67], plays a role in explaining how night-time SMU disrupts sleep. Active engagement in media activities can directly induce physiological and psychological arousal, leading to longer sleep onset latency [68]. This effect is particularly noticeable when individuals actively engage in interactive digital media, such as social messaging and social media, as opposed to passive media consumption like television viewing [69•], likely due to the heightened arousal associated with interactive activities [70]. Interestingly, a study found that engaging in phone conversations near bedtime was associated with longer sleep duration, while the use of social media and texting displayed a negative association [71]. It has been hypothesized that conversing with a friend may positively influence emotional well-being, thereby promoting sleep [72]. However, social networking, despite its potential for fostering friendships, may also trigger FOMO and social media stress. In addition to the psychological arousal induced by electronic media usage, the light emitted from device screens is another hypothesis explaining the detrimental effects of digital media on sleep. Specifically, the light emitted by electronic devices, especially blue light (at a wavelength of 480 nm), has a significant impact on the suppression of melatonin, a hormone that promotes sleep [73]. Moreover, a recent study indicated that high-risk adolescents whose parents with bipolar affective disorder have lower level of nocturnal melatonin secretion. It might be possible that adolescents with certain risk factors (even without psychopathologies) may be particularly hypersensitive and vulnerable to light suppression of melatonin secretion [74], which are considered as high-risk group that require early intervention. Furthermore, it is plausible that an interaction or interplay might exist between arousal and light exposure, and the combination of these conditions could potentially heighten the risk of sleep disturbances. Additionally, the direct displacement of sleep resulting from engagement in social media activities may also lead to shorter sleep duration. Although initial evidence suggests a negative impact of both content and light emitted by electronic devices on sleep, the precise underlying mechanism remains poorly established. Last but not least, some preliminary studies have also investigated other sleep- and circadian-related factors such as chronotype preference and daytime sleepiness in mediating and/or moderating the relationship between SMU and sleep [32•, 44, 54], albeit the findings have been inconclusive. Future studies are warranted to thoroughly explore the role of these factors in the interplay between SMU and sleep.

### Underlying Mechanism Between SMU and Mental Health

It has been suggested both behavioral and cognitive factors mediate the impact of SMU on mental health. Among the behavioral factors, sleep has been identified as one of the notable mediators of the association [31, 44, 45•, 46, 50, 59, 75]. Physiologically, prolonged SMU before bedtime delays sleep onset, reduces sleep duration, and mediates the association between eveningness and sleep as well as daytime sleepiness [44], which have been identified as risk factors for mental illness [76–78]. This complex interplay between sleep and mental health has also been documented in interventional studies. Our previous clinical trial demonstrated that a brief insomnia prevention program, adapted from cognitive-behavioral therapy for insomnia, significantly decreased the severity of depressive symptoms in adolescents at 12-month follow-up [79], suggesting the potential mediating role of sleep in mental health. While for the cognitive factors, FOMO has been recognized as a possible mediator. In particular, Elhai et al. reported that FOMO mediated relationship between anxiety and smartphone use frequency, as well as problematic SMU [80]. Besides FOMO, recent literatures have also identified several other cognitive factors that mediate the relationship between SMU and mental health, such as self-esteem [38•], body satisfaction [57], and emotional investment [59].

In addition to these behavioral and cognitive factors, cyberbullying is also one of the important mediators of SMU and mental health in youth [81]. Cyberbullying has become a prevalent phenomenon worldwide, with victimization rates in children and adolescents ranging from 14 to 57.5%, and lifetime perpetration rates ranging from 6.0 to 46.3% [82, 83]. Previous meta-analytical study demonstrated that cyberbullying significantly increased the risks of developing depression, self-harm, suicidal attempts, and ideation [84]. Moreover, over the COVID-19 pandemic, stressors associated with disasters have also been reported to potentially exacerbate the negative effects of SMU on mental health, thereby increasing the risk for mental health issues [85, 86].

In summary, there have been significant developments in recent years in understanding the magnitude and mechanisms that underlie the association between SMU and mental health. However, most of the studies employed a cross-sectional design, which prevented from a thorough understanding of the causality. Experimental and interventional studies are warranted to better comprehend the underlying mechanisms, establish causality, and improve the negative outcomes.

## Future Direction

There are several potential avenues for future investigation. Firstly, prospective cohort studies using representative samples are needed to elucidate the magnitude and directionality of relationships among SMU, sleep, and mental health, which is of clinical practice implication for precision intervention. To capture the varying dynamics among SMU, sleep, and mental health across different age groups of adolescents (early and late adolescents), it is recommended that prospective studies may need to have a follow-up period that will better cover the entirety of adolescence period [41]. Secondly, the lack of consistency in the methodologies employed by different studies measuring SMU has been a major contributing factor to the conflicting findings found in the current literature. It is imperative to use validated questionnaires to measure the SMU. More importantly, objective measurements (e.g., screen time monitors on smartphones [87], ecological momentary assessments [88], and wearable devices [89]) should also be incorporated. Apart from timing, it is equally important to capture the content, and number of devices that subjects engage with. Thirdly, future research should consider conducting randomized controlled trials at different levels (e.g., individual, school, and family) to reduce overall and problematic SMU and to ultimately improve sleep and mental health outcomes in youth. The design of the intervention may benchmark to existing guidelines such as the American Academy of Paediatrics recommendations 2016 on media use [90], and the WHO guidelines on physical activity and sedentary behavior [91], in which both guidelines recommend limiting the amount of recreational screen time, and avoiding SMU 1 h before bedtime. Intervention formats may consider psychoeducation, cognitive and behavioral techniques, and motivational interviewing. Finally, priority may be given to conduct observational and interventional studies on SMU in vulnerable populations, such as youth experiencing mood or sleep problems, as well as those who are high-risk offspring of parents with sleep and mood disorders, as these populations are more susceptible to experience significant negative impacts from inappropriate and excessive SMU [92].

## Conclusion

Despite the heterogeneity observed in the recent studies, both cross-sectional and cohort studies highlight the impact of SMU on poor sleep and mental health, albeit there are some inconsistent findings. Research has progressed from focusing solely on “screen time” to exploring the social, emotional, and cognitive dimensions of SMU. When measuring sleep outcomes, researchers have investigated the sleep duration and quality and also consider factors such as chronotype and pre-sleep arousal, which will enable a better understanding of how social media impacts sleep in a broader context. Similar advancements have also been made in the field of SMU-related mental health research. Recognizing the interconnections among SMU, sleep, and mental health is crucial for public health and will contribute to improving sleep and mental health outcomes while promoting rational SMU. Future studies should evaluate the effectiveness of interventions on reducing SMU, with ultimate goal to improve sleep and mental health.

## Data Availability

Since this review article solely relies on published articles and does not include individual participant data, therefore no data sharing is available.
